# Taking stock: protocol for evaluating a family planning supply chain intervention in Senegal

**DOI:** 10.1186/s12978-016-0163-7

**Published:** 2016-04-21

**Authors:** Francesca L. Cavallaro, Diane Duclos, Rebecca F. Baggaley, Loveday Penn-Kekana, Catherine Goodman, Alice Vahanian, Andreia C. Santos, John Bradley, Lucy Paintain, Jérémie Gallien, Antonio Gasparrini, Leah Hasselback, Caroline A. Lynch

**Affiliations:** Department of Infectious Disease Epidemiology, London School of Hygiene & Tropical Medicine, Keppel Street, London, WC1E 7HT UK; Department of Global Health and Development, London School of Hygiene & Tropical Medicine, 15-17 Tavistock Place, London, WC1H 9SH UK; Department of Disease Control, London School of Hygiene & Tropical Medicine, Keppel Street, London, WC1E 7HT UK; Management Science and Operations, London Business School, Regent’s Park, London, NW1 4SA UK; Department of Social and Environmental Health Research, London School of Hygiene & Tropical Medicine, 15-17 Tavistock Place, London, WC1H 9SH UK; Integrated Health Office, USAID Mozambique, Av Kenneth Kaunda, 193, Maputo, Mozambique

**Keywords:** Family planning, Supply chain distribution, Impact evaluation, Study protocol, Performance-based contracting, Senegal

## Abstract

**Background:**

In Senegal, only 12 % of women of reproductive age in union (WRAU) were using contraceptives and another 29 % had an unmet need for contraceptives in 2010–11. One potential barrier to accessing contraceptives is the lack of stock availability in health facilities where women seek them. Multiple supply chain interventions have been piloted in low- and middle-income countries with the aim of improving contraceptive availability in health facilities. However, there is limited evidence on the effect of these interventions on contraceptive availability in facilities, and in turn on family planning use in the population. This evaluation protocol pertains to a supply chain intervention using performance-based contracting for contraceptive distribution that was introduced throughout Senegal between 2012 and 2015.

**Methods:**

This multi-disciplinary research project will include quantitative, qualitative and economic evaluations. Trained researchers in the different disciplines will implement the studies separately but alongside each other, sharing findings throughout the project to inform each other’s data collection. A non-randomised study with stepped-wedge design will be used to estimate the effect of the intervention on contraceptive stock availability in health facilities, and on the modern contraceptive prevalence rate among women in Senegal, compared to the current pull-based distribution model used for other commodities. Secondary data from annual Service Provision Assessments and Demographic and Health Surveys will be used for this study. Data on stock availability and monthly family planning consultations over a 4-year period will be collected from 200 health facilities in five regions to perform time series analyses. A process evaluation will be conducted to understand the extent to which the intervention was implemented as originally designed, the acceptability of third-party logisticians within the health system and potential unintended consequences. These will be assessed using monthly indicator data from the implementer and multiple ethnographic methods, including in-depth interviews with key informants and stakeholders at all levels of the distribution system, observations of third-party logisticians and clinic diaries. An economic evaluation will estimate the cost of the intervention, as well as its cost-effectiveness compared to the current supply chain model.

**Discussion:**

Given the very limited evidence base, there is an important need for a comprehensive standardised approach to evaluating supply chain management, and distribution specifically. This evaluation will help address this evidence gap by providing rigorous evidence on whether private performance-based contracting for distribution of contraceptives can contribute to improving access to family planning in low- and middle-income countries.

**Electronic supplementary material:**

The online version of this article (doi:10.1186/s12978-016-0163-7) contains supplementary material, which is available to authorized users.

## Plain english summary

Approximately one-third of women in Senegal who want to control the size of their family have no access to a modern contraceptive. One reason that women may not get the contraceptives they need is because of stock-outs at health facilities. Very little is known about the causes of stock-outs, and even less about how supply chains could be improved to ensure contraceptives are in health facilities when women need them.

This protocol describes an evaluation of a programme that uses private companies, under performance-based contracts, to deliver contraceptives from regional levels in Senegal directly to health facilities. The evaluation is one of the most comprehensive of its kind drawing on expertise of anthropologists, epidemiologists and health economists.

Effectiveness of the programme will be measured using annual surveys being undertaken at household and health facility levels and routine stockcard data from health facilities. Successful elements of the programme will be identified through a process evaluation that will include in-depth interviews with healthworkers, observations of personnel involved in the programme and diaries completed by nurses in facilities. An economic evaluation will be used to measure the full cost and cost-effectiveness of the programme. Finally, we will explore methods by which to estimate the effect of the supply chain programme separately from other Family Planning programmes being implemented at the same time.

This evaluation will contribute knowledge to the very limited evidence base for the impact of supply chain programmes on contraceptive availability and uptake.

## Background

Fertility has declined over the past several decades alongside an increase in contraceptive use in most low- and middle-income countries (LMICs). However, the pace of fertility decline has been slower in sub-Saharan Africa, where modern contraceptive use remains low relative to other world regions [1] and where an estimated 25 % of married women have an unmet need for family planning (FP) - the highest regional unmet need worldwide [2]. Among multiple barriers to accessing contraception, there is evidence to suggest that public facility stock-outs of FP commodities are common in some LMICs [3–6]. As a result, there is increasing interest in supply chain (SC) interventions. Unless otherwise specified, “FP” and “contraceptives” refer to modern methods, and “facilities” imply public facilities throughout this article.

**Fig. 1:**
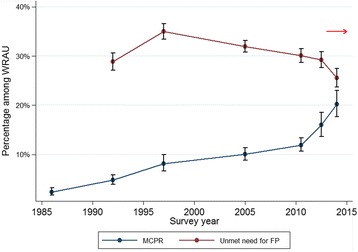
Modern contraceptive prevalence rate and unmet need for family planning among women of reproductive age in union (WRAU) over time. Source: Demographic and Health Surveys [48]. Note: The red arrow denotes start of intervention rollout across regions (early 2013: Dakar, Thies and Kaolack regions; 2014: Diourbel, Kaffrine, Fatick, Saint-Louis, Matam and Louga regions; 2015: Tambacounda, Kedougou, Kolda, Sedhiou and Ziguinchor regions)

In Senegal, only 12 % of women of reproductive age in union (WRAU) were using FP and another 29 % had an unmet need for FP in 2010–11 (Fig. 1) [7]. This prompted the Ministry of Health to announce a new strategy for reproductive health with the aim of increasing the modern contraceptive prevalence rate (MCPR) to 27 % by 2015 [8]. Low contraceptive use was thought to be partially driven by poor contraceptive stock availability in health facilities [9]. Both the government and development partners have made considerable investments in several FP initiatives, key among which is a supply chain model designed to reduce stock-outs called the “Informed Push Model”.

Supply chain management typically includes four main components: supply contract management; procurement; inventory management; and logistics and distribution. In most LMICs, supply chain systems for medicines are dominated by the Central Medical Stores model [10]. This model is both administratively and physically centralised, with procurement typically taking place at the national level, beyond which supply chains usually follow a country’s administrative structures, irrespective of country or population size [11].

“Push” and “Pull” are two primary mechanisms by which to distribute products to peripheral levels. A push system is one in which a central or regional storeroom determines the quantities of medicines to be ordered nationally and issued to each lower-level facility, based on population estimates, previous demand, and stock availability at the central level. While a push system can lead to more equitable rationing in the event of stock scarcity, it can result in stock-outs and wastage of products if forecasting at the central level does not take into account variation in need at the peripheral level. In contrast, a pull system is based on consumption data (demand) to determine the stock quantity to be ordered from a higher-level storeroom, thereby relying on decision-making at lower levels of the system. Pull systems are thought to allow for a more efficient distribution of medicines within the supply chain system, reducing the quantity of standing stock at any time and therefore of product wastage.

In many countries the supply chain is more complex than suggested by these models, with numerous actors at each level of the distribution system. Contraceptive supply chain systems in sub-Saharan Africa face a number of challenges, including poor infrastructure, inadequate forecasting, insufficient funds, delays in funding disbursement, long lead times, and political instability [6]. These challenges are compounded by the complex structural and institutional arrangements governing the distribution of medicines [12, 13], such as the presence of multiple donor-funded, programme-specific supply chains (such as for anti-malarial therapy or vaccines) running parallel to the national supply system. For example, in Senegal in 2009, the public sector supply chain had 11 main categories of health products at the central level, procured using 13 different sources of funding, through 12 different procurement organisations [14]. Despite these challenges, the performance of supply chain distribution is rarely monitored in-country. Periodic surveys indicate that FP stock availability is poor in public sector facilities in many countries. In 2013, UNFPA reported that an average of 55 % of facilities reported at least one contraceptive stock-out for any method in the previous 6 months in 12 countries in sub-Saharan Africa, ranging from 0.5 % in Ethiopia to 96.7 % in Cote d’Ivoire [6].

### Evidence on supply chain interventions in low- and middle-income countries

Multiple supply chain interventions have been piloted in LMICs in the last decade, with the aim of addressing gaps in the supply chain and improving availability of medical commodities at peripheral health levels [15–21]. However, very little robust evidence exists on the effect of different supply chain models or their sustainability without external support. Most studies examining the effects of alternative models on stock availability have been relatively small-scale pilots, and use poorly defined or different stock-out indicators, limiting the comparison of findings [22]. Only two models have been evaluated at the national level for essential medicines (including contraceptives) in sub-Saharan Africa, in Zambia [23] and Zimbabwe [24], though findings have not been peer-reviewed.

In Zambia, the 2006 Public Expenditure Tracking Survey found that essential and life-saving medicines were largely unavailable in facilities throughout the country [25], and a subsequent survey reported that progesterone-only pills, injectables, implants and IUDs were unavailable in at least half of facilities in four regions in 2008 [3]. In 2009, two new distribution models (A and B) were compared to the current supply chain system (model C), which relied on districts to deliver supplies to health facilities [26]. In model A, drugs were ordered at the district level with the support of a trained commodity planner. In model B, health facilities ordered directly from the national stores, where pre-packaged orders for facilities were delivered to the district level for onward distribution. In districts implementing model B, stock-out levels for injectables were reduced from 45 % of facilities during the 2-month baseline period to 1 % in the endline period; in districts implementing model A, these figures were 38 and 17 %, respectively [23]. Crude results were not reported for model C, but the difference-in-difference regression results suggest that stock-outs increased in districts implementing model C in the same period. The intervention models, by their design, included additional supervision of, and contact with, health facilities, making it difficult to determine whether the results can be attributed to the supply chain model or to increased communication with facilities.

In Zimbabwe, the Zimbabwe Informed Push (ZIP) uses delivery teams to perform contraceptive stock inventories in in health facilities, and top up stock levels to a pre-determined quantity [27]. The “push” component of the model derives from the fact that ordering and stock management is done by regional teams rather than facilities. Piloted in 2003 in two provinces, condom stock-outs were reduced from 20 % of all facilities to 2 % after the introduction of ZIP [27], though the timeframe used in this measure is not clearly defined. In 2007, an evaluation reported that the system had achieved 99 % coverage of all health facilities and ~95 % availability of contraceptives and condoms [27].

Supply chain interventions are complex, interfacing with the health system at multiple points and involving a number of components that can be adapted to a specific country context. Yet little detail is available in the literature describing specific components of interventions and their implementation, such as training or information systems. Little is known about the acceptability and sustainability of alternative supply chain models, particularly in regards to the introduction of new stock management personnel, or unintended consequences resulting from adapting supply chain models to different contexts. Relatedly, few studies have investigated the cost of supply chains, their cost-effectiveness, or their financial sustainability in comparison to alternative or current distribution systems [24, 28, 29].

Finally, there is uncertainty regarding the impact of improved stock availability on contraceptive use. The FP supply environment [30, 31], contraceptive logistics system performance [32] and an increase in the number of FP methods available at health facilities [32, 33] have been shown to have a positive effect on the MCPR. Wang *et al* [30] found a positive association between contraceptive use and increased availability of methods in health facilities close to where women were surveyed in four East African countries. Local informal networks of women sharing information and experience of FP are thought to be an important determinant of contraceptive behaviour [34, 35], and may be a mechanism through which improved contraceptive stock in facilities would translate into increased FP use. However, to our knowledge no study to date has examined the change in MCPR following a contraceptive supply chain intervention empirically, though there have been efforts to model this population-level impact (such as the Reducing Stockouts Impact Calculator [36]). Given the very limited evidence base relating to both the effect of supply chain interventions on stock availability and their impact on contraceptive use, there is an important place and need for a comprehensive standardised approach to evaluating supply chain management, and distribution specifically.

### Design of the intervention – “the Informed Push Model”

The health system structure in Senegal follows a hierarchical model, with procurement centralised at the national level. Each of the 14 regional medical offices is responsible for overseeing the health districts within the region. Health districts typically include one health centre in the main town, as well as a network of predominantly rural health posts [37].

**Fig. 2:**
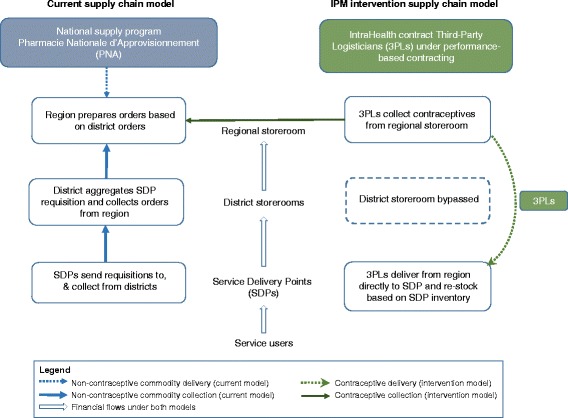
Comparison of current supply chain model (non-contraceptive commodities) and intervention supply chain model (contraceptives). SDP: Service Delivery Point; 3PL: Third-Party Logistician

*The current supply chain model* (Fig. 2) is used to distribute most non-contraceptive medical commodities in Senegal, and is run by the central Senegalese supply programme (Pharmacie Nationale d’Approvisionnement, or PNA). Public Service Delivery Points (SDPs, i.e. health centres and health posts) submit stock requests to the district, which sends an aggregate order to the regional storeroom. Regional storerooms send aggregate orders to the national supply program, and after receiving supplies, prepare them for collection by the districts. Each SDP is then responsible for collecting their order from the district storerooms.

*The intervention supply chain model* (also shown in Fig. 2) is being implemented by non-profit organisation IntraHealth International with funding from the Gates Foundation and MSD for Mothers, with the aim of improving the availability of FP commodities at public SDPs throughout Senegal. Private companies are contracted as third-party logisticians (3PL) to deliver FP commodities from regional storerooms directly to SDPs, bypassing the district level (thereby level-jumping). 3PLs make monthly deliveries to SDPs, during which they perform a stock inventory, top up contraceptive stocks to a level calculated based on consumption (around 3 months of stock), and collect data to forecast future delivery quantities. 3PLs are contracted by the implementer under fixed-fee performance-based contracts, with incremental penalties based on stock-out levels, re-supply delay and data availability [9]. Quarterly audits of 10 % of SDPs are conducted by the implementer to verify 3PL performance.

Eight products were initially included for distribution in the intervention (the combined pill, progesterone-only pill, injectable, implant, intra-uterine device (IUD), cycle beads, and male and female condoms), to which emergency contraception and a new sub-cutaneous injectable (Sayana Press) were added later. The initial stock of contraceptives is provided to SDPs at no cost, thus SDPs pay in arrears only for products sold, rather than paying upfront as in the current supply chain model [9]. A margin from the sale of FP commodities to women is retained at the SDP, district and regional levels, and 50 % of the margin is retained at the national level.

**Fig. 3:**
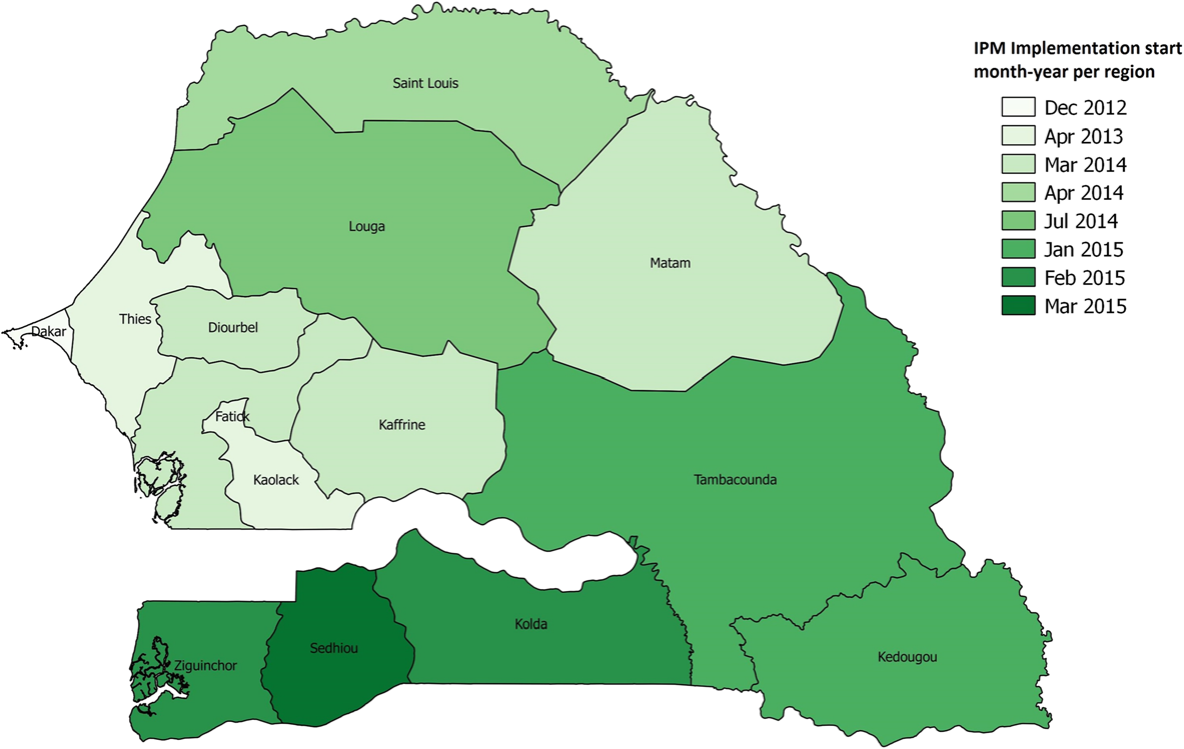
Map of Senegal with intervention rollout dates

A pre-intervention study in 2011 found that injectables were stocked out on 43 % of days and implants on 83 % of days in two districts in Dakar [9]. Subsequently, the intervention was piloted in one of these districts for 6 months in 2012. Stock-outs of contraceptive pills, injectables, implants and IUDs were reported to be eliminated in all 14 public SDPs in the pilot district, while they persisted at an average of 23 % of days in the comparison district [9]. After the pilot, the intervention was implemented throughout Dakar, where it achieved stock-out levels below 2 % of days within 6 months, and in Kaolack and Thiès regions in early 2013. It was eventually rolled out to the remaining 11 regions in a staggered manner between 2013 and March 2015 (Fig. 3). An adapted version was implemented in Saint Louis region where the distribution from regional to SDP level was implemented by the national supply programme, rather than outsourced to a 3PL.

### Intervention theory of change

**Fig. 4:**
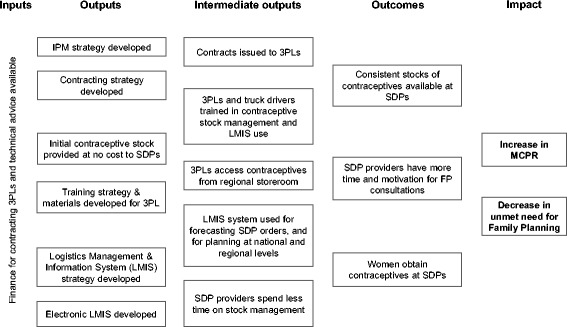
Theory of Change for the intervention (Senegal Informed Push Model). FP: Family planning IPM: Informed Push Model (intervention); SDP: Service Delivery Point; 3PL: Third-Party Logistician

A theory of change for the intervention was developed by the study team in collaboration with the implementer in early 2014, with details of how the intervention inputs and outputs were expected to lead to improved outcomes and impact (Fig. 4). An expanded version of the theory of change is presented in Additional file 1. Though a simplified model, the theory of change provides a useful framework to understand how the intervention was designed to work. Two main pathways are thought to help increase contraceptive stock availability and contraceptive use: first, performance-based contracting of 3PLs will ensure a consistent supply of contraceptives in SDPs. Second, the stock reporting system will allow for accurate forecasting of demand, avoiding both stock-outs and wastage at all levels of the system.

Two key assumptions underlie the success of the intervention if implemented as designed. The first is that performance-based contracting for 3PLs is effective in ensuring a consistent supply of contraceptives at SDPs. There are two main components to this intervention: performance-based contracting and outsourcing to private companies. A recent Cochrane review investigating the impact of pay-for-performance among health care providers in LMICs found that the current evidence base was too weak to make firm conclusions [38], and performance-based contracting for distribution of medical commodities has similarly not been studied extensively. Similarly, a review of the impact of contracting out health services on health care use and health outcomes identified three studies suggesting that contracting out can help increase access to health services, though these conclusions could not be generalised due to study weaknesses [39]. It is therefore unclear whether performance-based contracting of 3PLs can improve stock availability in Senegal. The second major assumption that improved availability of contraceptives in SDPs will lead to an increase in FP use by women may be valid if poor stock availability is a major barrier to use. However, multiple factors contribute to low contraceptive use in a context of high unmet need for FP, including distance to SDPs, financial barriers, lack of social acceptance of contraceptive use, as well as knowledge of and attitude towards FP [40, 41].

### Objectives of the evaluation

The broad aims of this study are to evaluate the effect of the intervention on stock availability and contraceptive use in Senegal, and to understand the acceptability, cost and cost-effectiveness of the intervention, as well as the context within which it was implemented. The four objectives of this evaluation are to:

1. Determine the effect of the intervention on contraceptive stock availability in SDPs and on contraceptive use among women
2. Assess the extent to which the intervention was implemented as designed, how it was modified during implementation, and how 3PLs functioned within the health system under performance-based contracts
3. Describe how contextual factors influenced the effect of the intervention on contraceptive use
4. Estimate the cost of the intervention and its cost-effectiveness compared to the current supply chain distribution model.

Throughout the evaluation, equity and sustainability will be cross-cutting themes of the different studies. An outcome and impact evaluation will be used to address objective 1, a process evaluation for objective 2, and an economic evaluation for objective 4. Objective 3 will be addressed using exploratory qualitative and quantitative methods to quantify the implementation intensity of FP interventions and quality of FP services in Senegal. A collaboration will be established with a multi-disciplinary team in Dakar. Trained researchers in qualitative, quantitative and economic research methods will implement the studies separately but alongside each other, sharing findings throughout the project to inform each other’s data collection. The specific objectives and mix of methods for each sub-study are described in turn.

## Methods

### Study setting

Senegal is a West African country with a population of approximately 14 million. It is classified as a lower-middle income country, with a Human Development Index rank of 163 out of 187 countries in 2013 [42]. Reproductive data are available from the 2014 Demographic and Health Survey [43], however we use data from the 2012–13 survey here to describe the context at the start of the intervention implementation. In Senegal, half (47 %) of women aged 25–29 were married at age 18 or below in 2012–13 [44]. The total fertility rate was 5.3 children per woman, with an average of 4.1 for women living in urban areas and 6.3 in rural areas [45]. Following the slow rise in modern contraceptive use reaching 12 % of WRAU in 2010–11, the MCPR increased to 16 % by 2012–13 (Fig. 1). An estimated 29 % of WRAU still had an unmet need for FP in 2012–13, implying that almost one third of women in union who did not want to get pregnant within the next 2 years were not using contraception [45].

Sharp differences existed in the MCPR between urban and rural areas (27 % compared to 9 %, respectively) and across educational levels (29 % among WRAU with secondary education and above, compared with 12 % among WRAU with no formal education). The most popular contraceptive method was injectables, accounting for 39 % of modern contraceptive users, followed by the pill (32 %) and implants (17 %) [45]. The public sector supplied the vast majority of contraceptives, with 83 % of modern contraceptive users obtaining their last method in a public SDP, and above 90 % for injectables and IUDs. The private sector supplied 13 % of users (predominantly condoms and pills) [44].

### Outcome and impact evaluation (Objective 1)

Two sub-objectives will be addressed in the impact evaluation: (1.1) evaluate the effect of the intervention on contraceptive stock availability in SDPs, and (1.2) examine the effect of changes in stock availability on the MCPR at the population level. Table 1 presents the key outcome and impact indicators used for objective 1, based on data collected by two studies.

**Table 1:** Indicators used for outcome and impact evaluation

Indicator	Data source
1. Stock availability in SDPs^a^	
Percentage of SDPs with all methods available on a given visit (%)	SDP survey; Service Provision Assessments [46]
Percentage of SDPs with each of the most popular short-, medium- and long-acting methods available on a given visit (%)	SDP survey; Service Provision Assessments
Percentage of the month/year stocked-out for any method (%)	SDP survey; Service Provision Assessments
Percentage of the month/year stocked-out for the most popular short-, medium- and long-acting methods (%)	SDP survey; Service Provision Assessments
Percentage of SDPs experiencing any contraceptive stock-out since intervention roll-out (%)	SDP survey
Average duration of stock-outs	SDP survey
2. FP^a^ consultations	
Monthly number of FP consultations	SDP survey
Monthly number of FP products distributed	SDP survey
Percentage of long-acting methods among monthly FP products distributed (%)	SDP survey
3. Contraceptive use	
Percentage of WRAU^a^ using a modern method^b^ of contraception (%)	Demographic and Health Surveys [48]
Percentage of single, sexually active WRA^a^ using a modern method of contraception (%)	Demographic and Health Surveys
Percentage of WRAU with an unmet need for FP (%)	Demographic and Health Surveys
Percentage of modern contraceptive users using a long-acting method^c^ (%)	Demographic and Health Surveys

^a^ *SDP* Service Delivery Point; *FP* family planning; *WRAU* Women of Reproductive Age in Union; *WRA* Women of Reproductive Age

^b^Modern methods include condoms, pills, injectables, implants, intra-uterine devices (IUDs), sterilisation

^c^Long-acting methods include implants and IUDs

The first study will follow a non-randomised stepped-wedge design using secondary data. To determine the impact of the intervention on contraceptive stock availability (objective 1.1) we will use data from Service Provision Assessments [46], nationally representative surveys of health facilities conducted annually in Senegal.

A general mixed-effects logistic regression model will be constructed using an approach similar to the stepped-wedge analysis method outlined by Hussey and Hughes [47], although the rollout was not randomised. Stock availability will be modelled as a binary outcome variable (stock present or absent on the day of the survey) for all contraceptive methods, and as an ordinal variable for the number of each of the most popular short-acting (condoms and pills), medium-acting (injectables) and long-acting methods (implants and IUDs). Time will be treated as a fixed effect, and region as a random effect, to allow the intervention effect to vary regionally. Independent covariates at regional level, such as density of SDPs offering FP services and density of road network, will be used as well as SDP-level variables such as facility type.

Similarly, to examine the effect of the intervention on contraceptive use among WRAU (objective 1.2) we will use information on contraceptive use at the population level from the Demographic and Health Surveys [48], nationally representative surveys of women of reproductive age also conducted annually. As above, a mixed-effects logistic regression model will be built treating region as a random effect, and including woman-level covariates (such as education level) and regional covariates (such as pre-intervention MCPR). For both objectives, secondary analyses allowing for a delay in the effect of the intervention will be explored by allowing the variable for the intervention mode to be fractional.

The second study will be a 4-year monthly time series of contraceptive stock availability (objective 1.1) and FP consumption (objective 1.2), constructed from data from a sample of SDPs. Five of the 13 regions where the intervention was implemented by 3PLs (i.e. excluding Saint Louis) will be randomly selected, and 10 districts randomly selected in each region with probability proportional to the number of SDPs in the district. In each selected district, the health centre will be included, as well as three randomly selected health posts, for a total sample of 200 SDPs across urban and rural locations. There is limited guidance on sample size calculations for time series analysis, which usually focuses on the number of time points rather than of sampling units [49]. At least 48 monthly observations on stock inventory and FP consultations will be collected from each SDP, as well as from the selected district and regional storerooms, and the national-level storeroom, ensuring at least 24 monthly time points pre- and post-intervention.

Information on contraceptive stock availability will be extracted from stock cards, daily patient registers and stock journals, as well as for several ‘tracer’ stocks (anti-malarials, oral rehydration salts, amoxicillin, and iron tablets) not expected to change as a result of the intervention. Monthly number of FP consultations in which patients receive a contraceptive at the SDP will be extracted from patient registers. A time series analysis will be conducted by constructing a generalised linear segmented regression model of the probability of stock-out for any method on a given month, and for the most popular short-, medium- and long-acting methods. We will examine whether there is a change in the slope or level of the number of methods available and monthly FP consumption at SDPs between the pre- and post-intervention periods. This analysis will be repeated for the tracer stocks to compare their change in stock availability with FP methods as a proxy measure of the ‘normal’ distribution channels through which these commodities are still distributed.

### Process evaluation (Objective 2)

The process evaluation will be informed by both qualitative and quantitative studies to gain a better understanding of what the intervention consisted of, the extent to which it was implemented as planned, and how well 3PLs functioned within the health system. Trained researchers in the different disciplines will implement the quantitative and qualitative studies separately but alongside each other, and the different teams will share findings throughout the course of the work to inform each other’s data collection.

Quantitative monthly indicator data from the implementer will be analysed to examine to what extent different components of the intervention were implemented as intended. The main indicators of interest will relate to timeliness of deliveries, achievement of stock-out rates below the 2 % target, and availability and use of data for informing stock orders (Table 2).

**Table 2:** Indicators used for process evaluation

Indicator	Data source
1. Intervention roll-out	
Percentage of SDPs^a^ receiving FP^a^ product through intervention (%)	Monthly data from implementing organisation
2. Timeliness of visits	
Percentage of 3PL^a^ visits conducted after scheduled date (%)	Monthly data from implementing organisation
Percentage of requests for emergency re-supply followed by unscheduled 3PL visit within 3 days (%)	Monthly data from implementing organisation
3. Stock-outs	
Percentage of 3PL visits with stock-outs observed (%)	Monthly data from implementing organisation
4. Data availability and use	
Percentage of SDPs with data for all 3PL visits (%)	Monthly data from implementing organisation
Percentage of SDPs with correct data for all 3PL visits compared to stock card information (%)	Monthly data from implementing organisation
Percentage of 3PL visits with complete information for date and products (%)	Monthly data from implementing organisation
Percentage of 3PL visits where the calculated quantity is delivered (calculated as three months’ average consumption minus stock available) (%)	Monthly data from implementing organisation

^a^ *SDP* Service Delivery Point; *FP* Family planning; *3PL* Third-party logistician

**Fig. 5:**
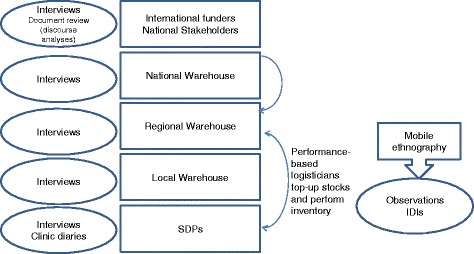
Data collection methods used in the ethnography of the supply chain

Qualitative data collection will be conducted at all levels of the health system (Fig. 5) by trained anthropologists, supervised by senior academics with in-depth knowledge of the context. A discourse analysis of funding documents, proposals and project reports related to the intervention will be conducted to refine the initial theory of change elaborated by the research team.

Repeat in-depth interviews will be conducted with key national and international stakeholders, during and immediately after the implementation period, to allow researchers to develop a rapport with interviewees, improve data quality and monitor change over time. These interviews will focus on the development of the project, implementation issues and lessons learned, to understand how closely the implementation followed the intervention design laid out in the theory of change. In-depth interviews will also be conducted with all cadres of personnel involved in the supply chain and FP at the regional, district and SDP levels (including SDP staff, programme auditors and 3PL), to examine the acceptability of performance-based contracting, how the intervention was modified in different contexts, and potential unanticipated issues. In-depth interviews will be administered using topic guides, which will be piloted and developed iteratively as data emerge.

Three repeat in-depth interviews will be conducted over the evaluation period with up to 10 key informants at the national level. In-depth interviews will be conducted with regional medical officers (up to 11), key implementing staff at the regional level (up to 15), 3PLs and auditors (up to 10), as well as with key stakeholders from the national (*n* = 1) and regional storerooms (*n* = 11), district stock managers (*n* = 11), health workers (*n* = 60) and pharmacists (*n* = 10). Up to 160 in-depth interviews will be conducted in total; the final sample size will follow the principle of saturation, wherein data collection continues until new data do not shed further light on the research questions. Sampling for interviews will be purposive to represent urban and rural areas and the diverse types of SDPs as well as distance from regional medical storerooms, and time since introduction of the intervention.

Ethnographic work will be carried out to understand the logics and practices of implementation on the ground. Researchers will travel with 3PL while performing deliveries and carrying out stock inventories, and observe the lived realities of both 3PL and SDP staff. Transcripts of interviews and field notes will be translated and independently coded by researchers in London and Senegal using qualitative software. Coding will be undertaken along key themes that are being explored, allowing for unexpected issues that emerge, and jointly discussed by the qualitative team to come to a consensus on major findings.

In addition, we will ask 25 SDP managers to fill in fortnightly reflective diaries on issues relating to the implementation, giving them a voice to identify unanticipated issues to the research team. Preliminary findings from the diary study will be fed back to SDP managers to validate our interpretation of their writings and to give them a further opportunity to discuss collectively their perspectives on the project. Similarly, findings from all objectives will be presented to key stakeholders at several points throughout the evaluation, in order to check the validity of results and elicit potential reasons for these.

### Assessment of contextual factors (Objective 3)

The purpose of this component of the evaluation is to understand what factors influence the effect of stock availability in SDPs on contraceptive use among women, using exploratory qualitative and quantitative methods. We hypothesise that FP uptake in areas with contraceptive availability would be higher in areas with high-quality FP services, where women have better geographical and financial access to contraceptive services, and with more FP-related activities (other than the intervention), such as demand-generation activities. Therefore, several approaches will be used to assess the quality of FP services provided in SDPs, describe women’s physical access to FP services, and estimate the implementation intensity of other FP­related activities in regions throughout Senegal, in order to examine whether the effect of the intervention varied according to these factors.

First, the quality of FP services will be assessed using data from the Service Provision Assessments [46], which collect information on choice of methods offered, observations of FP consultations and exit interviews with clients and providers. An index of FP service quality will be developed based on frameworks developed by Bruce, Mauldin and Ross [33, 50], as well as other existing indices [51–55]. The median quality of FP services across SDPs will be calculated for each region of Senegal, and any observed regional differences in the change in MCPR (objective 1) will be interpreted in light of regional FP quality category (high, medium, or low)..

Second, GPS information for all SDPs in Senegal will be obtained from the Ministry of Health, coordinates for regional storerooms taken during fieldwork and road shape files obtained from ESRI [56]. QGIS software [57] will be used to map SDPs, storerooms and roads. The 2013 census data [58] will be used to overlay age-specific fertility rates on SDP points, and buffer zones created to determine where women do and do not have access to SDPs providing FP, SDPs with FP stock available and SDPs with high quality FP services. These results will be used to examine whether changes in stock availability have had a larger impact on MCPR in regions where women have better access to FP services. In addition, in-depth interviews will be conducted with women to triangulate these quantitative analyses, with the aim of understanding the barriers they face in accessing FP, the quality of care they experience in SDPs, and what aspects women value when seeking care in public SDPs (cost, counselling, availability of methods etc.).

Third, indicators to measure implementation intensity of FP-related activities (other than the intervention) will be developed using Heidkamp et al’s “snapshot” approach [59]. These indicators will be informed by in-depth interviews with different FP actors in Senegal (see Process evaluation), and calculated based on policy documents and reports from these organisations for each region. Results from the impact evaluation (objective 1) will be stratified by region according to the intensity of other FP-related activities in order to examine whether the effect of the intervention on contraceptive use was stronger in regions with other FP programmes active at the same time as the intervention.

### Economic evaluation (Objective 4)

The purpose of the economic evaluation is to estimate the cost of the intervention (including costs related to capital goods, training, 3PL distribution, and audit teams), and its cost-effectiveness in relation to the current supply model. A micro-costing approach will be used to estimate the cost of the intervention and current supply chain models at national, regional, district, and SDP levels. Costs for 3PLs will be measured as the price paid for by the implementer. Costs will be estimated for the entire supply chain, retrospectively to capture costs pre­intervention, start­up costs (including initial training) and costs post­intervention.

A survey of SDPs and district, regional, and national storerooms will be conducted to measure costs. Among the SDPs included in objective 1, up to 80 SDPs will be randomly sampled to include a range of SDP sizes and rural/urban locations; the district and regional storerooms in which these SDPs are located will also be surveyed. A questionnaire will be administered to SDP and storeroom managers in order to record staff involved in supply chain management, time and resources spent collecting stock from higher-level storerooms, and intervention-related activities such as training. Financial and economic costs of the intervention and the current supply chain system will be collected through document reviews and account classification. The development of the survey tools will be informed by the in-depth interviews conducted during the process evaluation (objective 2) in order to ensure all costs are captured.

In addition, a time and motion study will be conducted to directly measure and cost time required to manage, operate and deliver the commodities to SDPs. Data on human resource utilisation and flow of services relating to the FP supply chain will be collected through interviews with SDP managers and 3PLs, and time spent on supply chain activities will be estimated using structured observations of SDP staff involved in supply chain activities and 3PLs and self-reported timesheets.

Financial and economic costs will be categorised as: training and start-up costs, capital costs (including storage and transport equipment) and recurrent costs (such as staff salaries and maintenance expenditure). Capital costs will be estimated by using current (replacement) costs for all capital goods used in a year, annualised over the expected duration of their working life using a discount rate of 3 % according to World Health Organisation guidelines [60].

The aim of the cost-effectiveness study is to compare the incremental costs and incremental effects of the intervention compared to the current supply chain model for contraceptives. Since the intervention has already been rolled out, the pre-intervention costs of distributing FP commodities will be estimated by calculating costs of the current distribution system for non-contraceptive ‘tracer’ commodities (anti-malarials, oral rehydration salts, amoxicillin, and iron tablets). Estimates for the incremental effectiveness of the intervention compared to the current supply chain model will be obtained from the impact evaluation (objective 1). Several effect measures will be used to calculate incremental cost-effectiveness ratios (Table 3). Data on stock availability in SDPs will be used to calculate FP consumption lost due to stock­outs and the cost per stock­out averted for all stock­outs and for the most commonly used short­, medium­ and long­acting methods.

**Table 3:** Indicators used for economic evaluation

Indicator	Data source
1. Cost measures	
Mean cost of delivery to SDPs^a^ and 3PLs^a^	SDP and district/regional/national storeroom survey and document review
Income from FP^a^ products sold	SDP and district/regional/national storeroom survey and document review
Income from FP consultation fees	SDP and district/regional/national storeroom survey and document review
2. Effectiveness measures	
Number of stock-outs averted for all products	SDP and district/regional/national storeroom survey and document review
Number of stock-outs averted for each of the most popular short-, medium- and long-acting product	SDP and district/regional/national storeroom survey and document review
Couple-years of protection provided	SDP survey and document review
Number of additional WRAU accessing modern contraception	SDP survey and document review, Demographic and Health Surveys [48]
Number of pregnancies averted	SDP survey and document review, Demographic and Health Surveys, and model parameters informed by maternal & reproductive health literature
Number of unsafe abortions averted	SDP survey and document review, Demographic and Health Surveys, and model parameters informed by maternal & reproductive health literature
Number of maternal deaths averted	SDP survey and document review, Demographic and Health Surveys, and model parameters informed by maternal & reproductive health literature

^a^ *SDP* Service Delivery Point; *3PL* Third-party logistician; *FP* Family planning; *MCPR*: modern contraceptive prevalence rate

Modelling techniques, in addition to assumptions derived from the literature, will be used to estimate the cost per couple­year of protection; cost per additional WRAU accessing modern contraception; and cost per pregnancy averted, unsafe abortion averted, and maternal death averted. In addition, the healthcare costs saved by averting pregnancies (such as costs of antenatal care, post­abortion care, pregnancy and birth complications) will be estimated. All cost estimates will be evaluated using probabilistic sensitivity analyses to identify the variables that had the largest impact on the model results.

The study design and data sources used to address the four main objectives are summarised in Table 4.

**Table 4:** Summary of study designs and data sources used to address each research objective

Research objectives	Study design	Data sources
1. OUTCOME AND IMPACT EVALUATION Determine the effect of the intervention on contraceptive stock availability in SDPs^a^ and on contraceptive use among women	Non-randomised study with stepped-wedge design of FP stock availability in SDPs	• National annual surveys of health facilities (SPA^b^) and women of reproductive age (DHS^c^)
	4-year time series of monthly FP^a^ stock availability and number of consultations in SDPs	• Survey of 200 SDPs and corresponding district, regional and central storerooms
2. PROCESS EVALUATION Assess the extent to which the intervention was implemented as designed, and how 3PLs^a^ functioned within the health system	Document and indicator review	• Funding documents and proposals • Project reports and monthly indicator data from implementer
	Ethnographic study of implementation, acceptability of 3PLs within health systems and potential unintended consequences	• Repeat in-depth interviews with key informants • Interviews with stakeholders at all levels of distribution system • Ethnographic study of 3PLs • Reflective implementation diaries by clinic managers
3. ASSESSMENT OF CONTEXTUAL FACTORS Describe how contextual factors affected the effect of the intervention on contraceptive use	Descriptive analysis of regional variations in quality of FP services	• National annual surveys of health facilities (SPA)
	Descriptive analysis of women’s access to SDPs	• Ministry of Health GPS coordinates for SDPs • National census data
	Assessment of implementation intensity of other FP-related activities by region	• In-depth interviews with FP actors in Senegal • Policy documents and reports from other organisations providing FP services
4. ECONOMIC EVALUATION Estimate the cost of the intervention and its cost-effectiveness compared to the current supply chain distribution model	Comparison of total cost of intervention and current distribution models	• Survey of 80 SDPs and corresponding district, regional and central storerooms • Review of funding documents
	Time and motion study of time spent managing, operating and delivering FP products to SDPs	• Interviews with clinic managers and 3PLs • Self-reported timesheets by SDP staff involved in supply chain activities
	Cost-effectiveness analysis	• Costs from the costing analysis and effects from the impact evaluation combined to estimate incremental cost-effectiveness ratios (ICERs)

^a^ *SDP* Service Delivery Point; *FP* Family planning; *3PL* third-party logistician

^b^Service Provision Assessments [46]

^c^Demographic and Health Surveys [48]

### Ethics, consent and permissions

Ethical approval for this study was obtained from the ethics committee of the Conseil National de Recherche en Santé (CNRS) in Senegal (n° 107/MSAS/DPRS/CNERS), and from the London School of Hygiene & Tropical Medicine (Ethics ref: 9925). Informed consent will be sought from participants for primary data collection-related activities, including in-depth interviews, structured observations and SDP diaries. For data extraction from SDPs and district, regional or national storerooms, consent will be sought from the head of the SDP or storeroom chief officer. Participants in qualitative data collection, and SDPs and storerooms, will be identified using a coded identifier, and the key will be stored securely in a password-protected file accessible to selected team members. In the reporting of the data, quotations will be anonymised to ensure individuals are not identifiable. GPS information for SDPs will be stored in a separate password­protected dataset. Audio files will be downloaded onto a password­protected computer hard drive and backed up regularly. All paper and soft copies of field notes will be kept in a locked cabinet and only shared within the study team.

## Discussion

We have set out a comprehensive framework for evaluating a FP supply chain model that aims to improve the availability of contraceptives in public SDPs, and ultimately, to increase contraceptive use and reduce unmet need for FP in Senegal. While a number of supply chain interventions are being piloted in LMICs, very little robust evidence exists to demonstrate the effect of such interventions on SDP stock-outs or MCPR. This framework is one that could be adapted and used elsewhere to evaluate the effect of a supply chain intervention on stock availability and, ultimately, health intervention coverage, outcomes and impact.

This evaluation will contribute comprehensive and robust evidence on the ability and cost-effectiveness of performance-based contracting of third-party logisticians to achieve consistent supplies of FP products at scale. Furthermore, using the theory of change approach in the process evaluation will yield a better understanding of what and how contextual factors affect the implementation of large-scale, complex interventions, as well as potential unintended consequences. This study takes advantage of, and is strengthened by, the availability of existing annual surveys of health facilities and women in Senegal to help verify data on stock availability over time collected from SDPs and implementing partners. Findings from the impact evaluation will further be triangulated using qualitative data collected through the process evaluation. The strength of the evaluation design combining process, impact and economic evaluation will help address the dearth of robust evidence on the effectiveness of supply chain interventions in health and inform Ministry of Health planning for the intervention continuation and improvement, as well as other countries piloting supply chain interventions.

There are several limitations to our approach. First, our ability to investigate contraceptive stock supply over time relies on the availability of good quality stock data in SDPs. Between 2010 and 2012, health staff in some regions of Senegal took strike action which manifested as a refusal to send health data to the district level, which may affect data availability for the time series study in regions where the 24-month pre-intervention period includes 2011–2012. Health facility and staff adherence to the strike was variable, and it is unclear whether stock data were particularly affected since a different cadre of staff (community-funded “dépositaires”, or stockists) is tasked with completing stock cards, rather than the centrally-funded nursing staff who were on strike. In addition, it is not clear whether, given the duration of the strike, data continued to be collected but were not sent upward or whether nursing staff and stockists stopped recording data in SDPs. The availability of good data in SDPs will be explored in the data quality pilot study prior to collecting data for the time series.

Second, estimating the contribution of the intervention to any observed increase in contraceptive use may be challenging given that other FP-related activities such as demand-generation activities, public endorsements of FP by community leaders, and outreach activities are implemented throughout the country. The evaluation methodology is designed to take this limitation into account as effectively as possible. The staggered rollout of the intervention across regions will enable us to estimate more precisely the effect attributable to the intervention. As part of the assessment of contextual factors (objective 3), we will further estimate the implementation intensity of FP-related activities in order to disentangle the potential effect of the intervention and of other FP-related activities on the MCPR.

Lastly, we will face challenges in evaluating an intervention that could potentially change over the course of the evaluation period in response to new challenges or to decisions by the Ministry of Health aiming to increase political will and ownership at national level. We have designed the evaluation to incorporate this by ensuring that we document changes to the intervention over time. In addition, the comprehensive nature of our approach, the implementation of continuous SPA and DHS surveys as well as the scale of both the intervention and the evaluation allows for us to evaluate changes in the overall context in Senegal even as changes are made to the supply chain intervention.

There is limited evidence on whether supply chain interventions can ensure sustained contraceptive availability in health facilities, and in turn increase contraceptive use, despite the growing number of supply chain pilots in LMICs. Robust and comprehensive evaluations of alternative distribution models are critically needed to understand whether they can improve access to contraceptives, and how they can best be implemented in order to reduce the unmet need for FP in LMICs. Thus, despite the challenges presented in evaluating a complex large-scale intervention, this evaluation will add important evidence to the field.

## Additional file

Additional file 1: Extended Theory of Change for the “Informed Push Model” in Senegal. Extended table with the full Theory of Change of intervention being evaluation in Senegal. (PDF 66 kb)

## Abbreviations

3PL: third party logisticians
ACT: artemisinin-based combination therapy
FP: family planning
GPS: global positioning system
LMIC: low- and middle-income country
MCPR: modern contraceptive prevalence rate
SDP: Service Delivery Point
WRAU: Women of Reproductive Age in Union
ZIP: Zimbabwe Informed Push
