# Supplementary figures and images for: Taking stock: protocol for evaluating a family planning supply chain intervention in Senegal
Source: Reprod Health. 2016 Apr 21;13:45. doi: 10.1186/s12978-016-0163-7 (PMC4839151; doi:10.1186/s12978-016-0163-7)

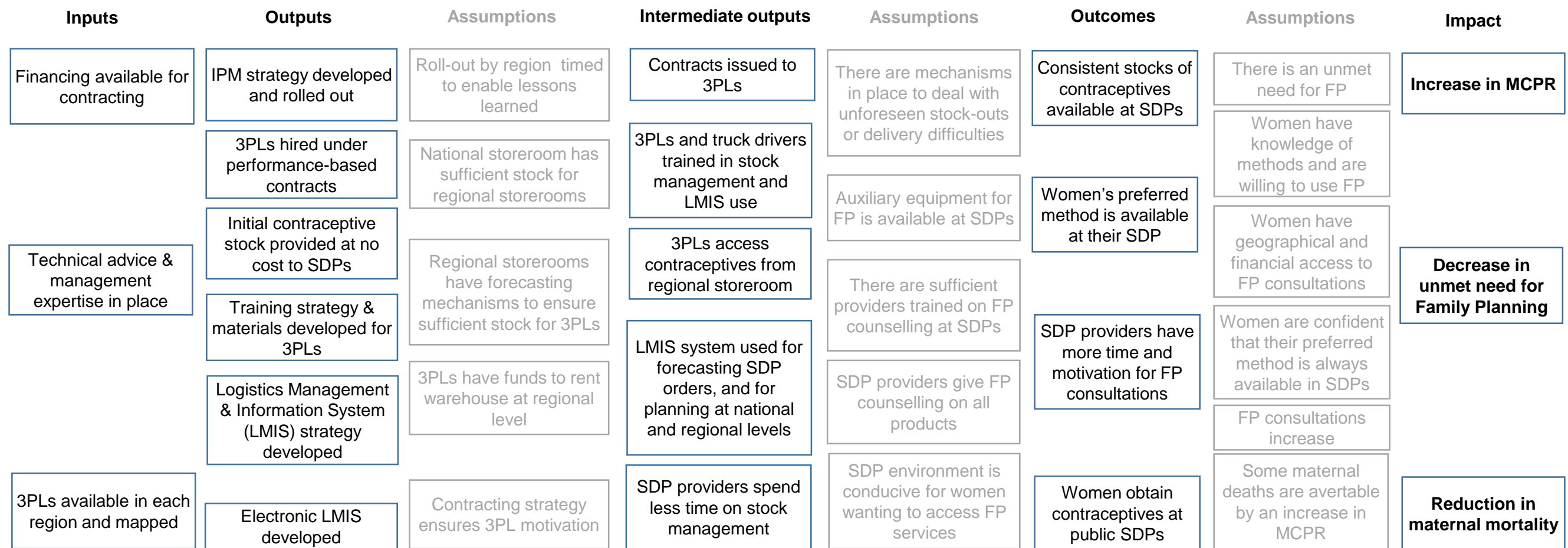

Supplement: Additional file 1: — Extended Theory of Change for the “Informed Push Model” in Senegal. Extended table with the full Theory of Change of intervention being evaluation in Senegal. (PDF 66 kb) [file 12978_2016_163_MOESM1_ESM.pdf]
